# *Reticulitermes nelsonae*, a New Species of Subterranean Termite (Rhinotermitidae) from the Southeastern United States 

**DOI:** 10.3390/insects3010062

**Published:** 2012-01-06

**Authors:** Su Yee Lim, Brian T. Forschler

**Affiliations:** Household and Structural Entomology Laboratory, Department of Entomology, 413 Biosciences Building, 120 Cedar St., University of Georgia, Athens, GA 30602, USA; E-Mail: bfor@uga.edu

**Keywords:** new species, taxonomy, phylogenetics, morphometrics, dichotomous key, Isoptera, Termitoidea

## Abstract

*Reticulitermes nelsonae*, a new species of Rhinotermitidae (Isoptera) is described based on specimens from Sapelo Island, GA, Thomasville, GA, Havelock, NC, and Branford, FL. Adult (alate) and soldier forms are described. Diagnostic characters are provided and incorporated into a supplemental couplet of a dichotomous key to the known species of *Reticulitermes* found in Georgia, USA.

## 1. Introduction

Members of the family Rhinotermitidae, commonly known as “subterranean termites”, have a cryptic lifestyle making them difficult to study [1,2]. In the United States, the genus *Reticulitermes* includes several economically notorious species that cause billions of dollars in structural damage every year [3,4,5,6,7]. Proper identification is critical to understanding the economic and ecological importance of these insects [8,9], and although there are four described species of *Reticulitermes* endemic to the southeastern United States, the current keys only address three [10,11,12]. Proper identification is complicated by intraspecific morphological variation which is characteristic of this genus as demonstrated by the quantitative measures that provide overlapping ranges for a number of characters for species collected in the United States [13,14,15,16]. The greatest diversity of *Reticulitermes* in the USA is in the eastern states where authors have suggested the presence of additional species [16,17,18,19,20,21,22].

Kollar (1837) [23] described the first extant member of the genus, *R. flavipes* from specimens found in Vienna, Austria, and this species was later found to be endemic to the eastern United States [10]. Three additional species were subsequently described from the eastern USA, including *Reticulitermes (Termes) virginicus* Banks 1907 [24], *R. hageni* Banks 1920 [10] and *R. malletei* Clément *et al.* 1986 [11]. Scheffrahn *et al.* (2001) [25] proposed that *R. malletei* was a *nomen nudum *yet Austin *et al.* (2007) [12] provided 16SrRNA sequence data and morphometrics supporting species status for *R. malletei* and showed that all nomenclatural requirements for the designation were met*. Reticulitermes santonensis *Feytaud 1924 [26] has been synonymized with *R. flavipes* [27,28]. Table 1 summarizes the taxonomic literature on *Reticulitermes* that mention the species endemic to the southeastern USA.

Herewith we provide a formal description for a new species collected in the southeastern USA, with diagnostic morphological characters and genetic corroboration. We also identify specific quantifiable morphological characters in combination with selected qualitative characters that are included in dichotomous keys to the soldier and alate of *Reticulitermes* species found in Georgia, USA.

**Table 1:** Summary of taxonomic literature that provide morphology and life history information on *Reticulitermes* spp. found in the southeastern United States, parenthetical letters are defined as follows: a = alate, s = soldier, species abbreviations are defined as follows: Rf = *R. flavipes*, Rv = *R. virginicus*, Rh = *R. hageni*, Rm = *R. malletei*, Ra = *R. arenincola*, Rhp = *R. hesperus*, Rt = *R. tibialis*.

Reference citation	Caste, notes	Pages	Species from USA
Banks, N. & Snyder, T.E. (1920)	(a, s) descriptions, illustrations, key, flight times	42–47, 148–164	Rf, Rv, Rh, Rhp, Rt
Goellner, E. J. (1931)	(a, s) descriptions, illustrations, ecology	227–234	Rf, Rt, Ra
Kofoid, C.A. (1934)	(a), descriptions, flight times	193–194	Rf, Rv, Rh, Ra, Rhp, Rt
Miller, E.M. & Miller, D.B. (1943)	(a, s) descriptions, illustrations, flight times	101–107	Rf, Rv, Rh
Banks, F.A. (1946)	(a, s) descriptions, illustrations, key, flight times	1–29	Rf, Rv, Ra, Rt
Miller E.M. (1949)	(a, s) descriptions, illustrations, key, flight times	6–7, 14–15, 20–22, 26	Rf, Rv, Rh
Snyder, T.E. (1954)	(a, s) descriptions, illustrations, key, flight times	26, 51–56	Rf, Rv, Rh, Ra
Miller E. M. (1964)	(a) flight times	5, 16	Rf, Rv, Rh
Weesner, F.M. (1965)	(a) descriptions, illustrations, key, flight times	36–44, 51	Rf, Rv, Rh, Ra, Rhp, Rt
Clément, J.L., Howard, R., Blum, M. & Lloyd, H. (1986)	(a, s) descriptions, life history	67–70	Rf, Rv, Rh, Rm
Nutting, W.L. (1990)	(a, s) descriptions, illustrations, key, flight times	997–1030	Rf, Rv, Rh, Ra, Rhp, Rt
Scheffrahn RH, Su N-Y. (1994)	(a, s) descriptions, illustrations, key, flight times	465–473	Rf, Rv, Rh
Hostettler, N.C., Hall, D.W. & Scheffrahn, R.H. (1995)	(s) descriptions, photographs	119–129	Rf, Rv, Rh
Ye, W., Lee, C.-Y., Scheffrahn, R.H., Aleong, J.M., Su, N.-Y., Bennett, G.W., Scharf, M.E., (2004)	(s) descriptions, photographs	815–822	Rf, Rv, Rh, Ra
Brown, K., Kard, B. & Payton, M. (2005)	(a, s) descriptions, photograph	277–284	Rf, Rv, Rh
Austin, J.W., Bagnères, A.-G., Szalanski, A.L., Scheffrahn, R.H., Heintschel, B.P., Messenger, M.T., Clement, J.-L. & Gold, R.E. (2007)	(a, s) descriptions, illustrations, photographs, flight times	1–26	Rf, Rv, Rh, Rm, Ra, Rhp, Rt
Wang, C., Zhou, X., Li, S., Schwinghammer, M., Scharf, M., Buczkowski, G. & Bennett, G. (2009)	(a, s) descriptions, key, photographs	1029–1036	Rf, Rv, Rh, Ra, Rt

## 2. Materials and Methods

### 2.1. Specimens

The morphological data for the new species were obtained from 96 soldiers, 141 alates, and 20 soldier mandible pairs. The number of soldier and alate specimens examined for the four previously described species ranged from 32 to 431 [29]. Genetic data for the new species were obtained from 156 specimens collected from Sapelo Island, GA (McIntosh Co.), Thomasville, GA (Thomas Co.), Havelock, NC (Craven Co.) and Branford, FL (Suwannee Co.).

All specimens were preserved in 70–100% ethanol. The number of specimens examined and collection information for the new species description are listed in Table 2.

**Table 2:** Collection information by site number, county (McIntosh Co. = Sapelo Island, Thomas Co. = Thomasville), sample size, and collection date by caste (s = soldier, a = alate) for specimens of *R. nelsonae* used for morphological measurements.

**Soldier**			
**Site ID**	**County**	**Sample Size**	**Collection Date**
Site 1s	McIntosh Co.	7	Nov 2007
Site 2s	McIntosh Co.	16	Feb 2007
Site 3s	McIntosh Co.	9	Feb 2007
Site 4s	McIntosh Co.	2	Feb 2007
Site 5s	McIntosh Co.	3	Feb 2007
Site 6s	McIntosh Co.	4	Feb 2007
Site 7s	McIntosh Co.	11	Feb 2007
Site 8s	McIntosh Co.	2	Jul 2009
Site 9s	McIntosh Co.	1	Jul 2009
Site 10s	McIntosh Co.	2	Jul 2009
Site 11s	McIntosh Co.	2	Jul 2009
Site 12s	Thomas Co.	1	Nov 2009
Site 13s	Thomas Co.	2	Nov 2009
Site 14s	Thomas Co.	4	Nov 2009
Site 15s	Thomas Co.	1	Nov 2009
Site 16s	Thomas Co.	2	Nov 2009
Site 17s	Thomas Co.	1	Nov 2009
Site 18s	Thomas Co.	2	Nov 2009
Site 19s	Thomas Co.	1	Nov 2009
Site 20s	Thomas Co.	2	Nov 2009
Site 21s	Thomas Co.	2	Nov 2009
Site 22s	Thomas Co.	1	Nov 2009
Site 23s	Thomas Co.	1	Nov 2009
Site 24s	Thomas Co.	1	Nov 2009
Site 25s	Thomas Co.	1	Nov 2009
Site 26s	Thomas Co.	1	Nov 2009
Site 27s	Thomas Co.	2	Nov 2009
Site 28s	Thomas Co.	3	Nov 2009
Site 29s	Thomas Co.	2	Nov 2009
Site 30s	Thomas Co.	2	Nov 2009
Site 31s	Thomas Co.	1	Nov 2009
Site 32s	Thomas Co.	1	Nov 2009
Site 33s	Thomas Co.	1	Jan 2010
Site 34s	Thomas Co.	1	Jan 2010
Site 35s	Thomas Co.	2	Mar 2010
Total = 35		N = 96	
**Alate**			
**Site ID**	**County**	**Sample Size**	**Collection Date**
Site 4a	McIntosh Co.	14	Feb 2007
Site 6a	McIntosh Co.	6	Feb 2007
Site 4a	McIntosh Co.	19	Mar 2007
Site 36a	McIntosh Co.	19	Mar 2007
Site 3a	McIntosh Co.	5	Feb 2007
Site 2a	McIntosh Co.	14	Feb 2007
Site 5a	McIntosh Co.	1	Feb 2007
Site 7a	McIntosh Co.	13	Feb 2007
Site 37a	McIntosh Co.	50	May 2005
Total = 9		N = 141	

**Figure 1:**
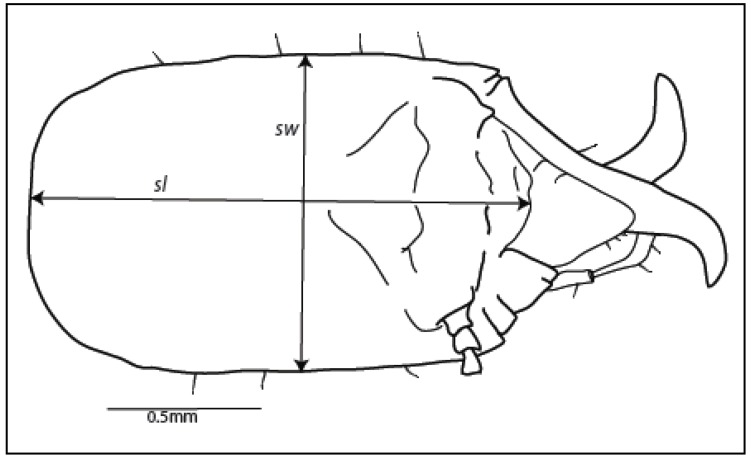
Standard measurements for soldier head capsule length (*sl*) and width (*sw*). Note that the length (*sl*) measurement does not include mandibles. Scale bar = 0.5 mm.

**Figure 2:**
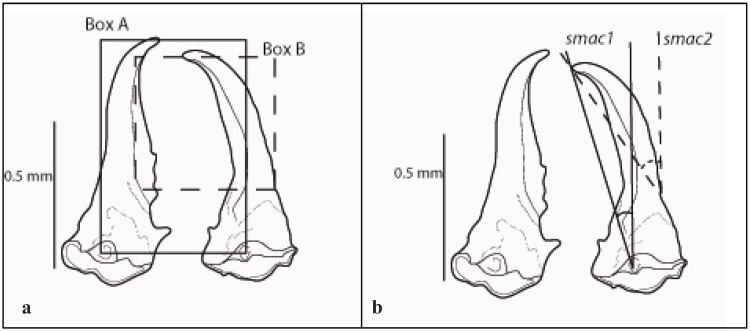
Diagram of soldier mandibles, dorsal view. Illustrating (**a**) the box method used to determine the 90° for measurement, (**b**) angles measured for *smac1* (Box A) and *smac2 *(Box B). Scale bar = 0.5 mm.

Accurate *Reticulitermes* species attributions are best determined using both soldiers and alates from the same collection site supported by genetic and behavioral information. The specific measurement points used for the quantitative dataset are shown in Figure 1 and Figure 2. 

### 2.2. Soldier

Four characters were measured on soldier head capsules: length without mandibles (*sl*), width (*sw*), and two separate angles of curvature for the right mandible (*smac1*, *smac2*). A fifth character, ratio of length:width (*sl:sw*), was calculated to determine its usefulness as a character for species separation.

Soldier head capsules were removed from the body and mounted by placing a minuten pin into the occipital foramen. The opposite end of the minuten was positioned into a cube of foam mounted on a standard size # 2 insect pin. Soldier head capsule length (*sl*) was measured from the clypeal sulcus to the posterior edge as seen from a dorsal view (near the occipital foramen), and width (*sw*) was measured at a 90° angle from the mid-point of *sl* (Figure 1). 

Soldier mandibles were dissected from head capsules and mounted on two-sided tape positioned inside a 2 mm × 2 mm grid box. Mandibles were positioned dorsal side up and parallel to the bottom line of the grid box to establish a 90° vertical line for the right mandible angle of curvature measurement (Figure 2a). The soldier right mandible angle of curvature (*smac*) was measured from two positions: the dorsal condyle (*smac1*) and external curvature inflexion point (*smac2*) (Figure 2b).

### 2.3. Alate

**Figure 3:**
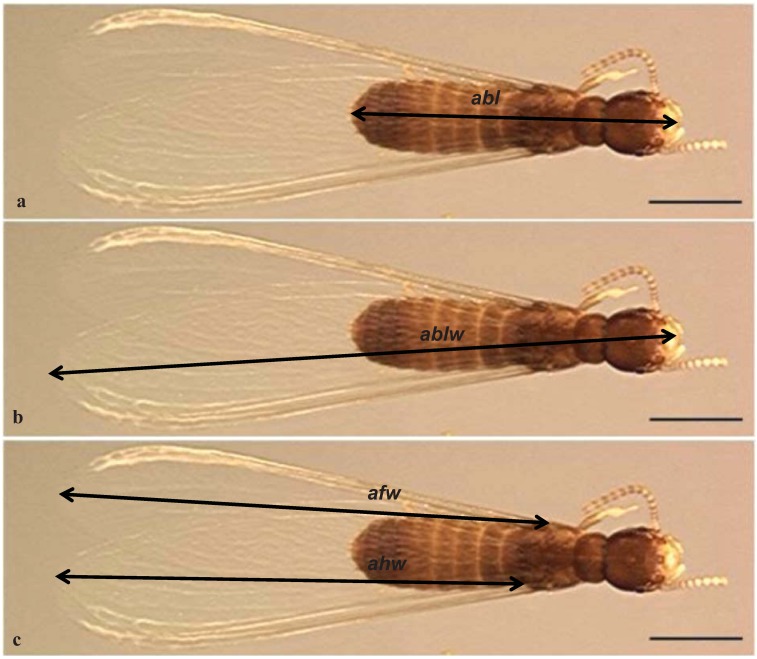
Standard measurements for alate characters: length of body only (*abl*) body-wing (*ablw*), wings (*afw* and *ahw*) measuremements. Scale bar = 1.0 mm.

Alates were mounted between a glass slide and cover slip in 100% ethanol [29]. Qualitative characters included body color (*abc*) and wing pigmentation (*awp*). Quantitative characters included body length (*abl*), body length including wings (*ablw*), average forewing length (*afw*) and average hind wing length (*ahw*) (Figure 3a–c).

### 2.4. Morphometrics, Imaging and Statistics

Soldier and alate specimen were prepared under a binocular dissecting microscope (CIT-OVAL2, Carl Zeiss aus Jena, Jena, Germany and Leica WILD M10, Wetzlar, Germany). Images were taken with a Sony DKC-5000 camera attached to a Leica WILD M10 stereomicroscope (Wetzlar, Germany) using Adobe Photoshop v.8.0 (Adobe Systems, San Jose, CA, USA). All soldier and alate images were taken at 25× and 20× magnification respectively, and calibrated with a micrometer using the internal preset calibration setting in AutoMontage Pro, v.5.0.1 (Cambridge, UK). Morphometric measurements were recorded using the AutoMontage Pro, v.5.0.1 and exported to Microsoft Office Excel (Redmond, Washington, USA). All statistical analyses of mean, standard deviation and simulation of sample size were performed using SAS v.9.2 (SAS Institute Inc., Cary, NC, USA), one-way analyses of variance (ANOVA) performed on each character state to determine if it contributed significantly to species separation. Sequential t-tests with LSD (protected least square deviation) pair-wise comparison were used to determine species differences for all characters measured. Step-Wise Discriminant Analysis (SWDA) was used to determine which morphological characters were most useful in species separation. The reliability and accuracy of each character state was determined using multiple Discriminant Function Analyses (DFA) [29].

### 2.5. Behavior

Fully developed, not-yet flown, winged alates of the new species were collected from infested wood on Sapelo Island on three separate dates.

### 2.6. Dichotomous Key

A dichotomous key for the *Reticulitermes* species of Georgia was constructed using morphological and behavioral (flight phenology) data. 

### 2.7. Molecular Data

Sequence data for two mtDNA genes obtained from workers, soldiers and alates were employed to provide additional support for the new species. Genomic DNA was extracted from selected specimens using either Promega’s Wizard Genomic DNA Purification Kit or Qiagen’s DNeasy Extraction Kit, following a modified protocol [22]. Primers used for amplification of the entire length of the mitochondrial COII and partial COI genes are listed in Table 3. Amplified PCR products were sequenced at Molecular Cloning Laboratories (South San Francisco, CA, USA) or Eurofins MWG Operon (Huntsville, AL, USA).

Sequences were curated with Sequencher 4.5 (Gene Codes Corp., Ann Arbor, MI, USA) and aligned with MUSCLE (MEGA 5 [30], or Phylogeny.fr: Robust Phylogenetic Analysis For The Non-Specialist [31]) using the default settings. Gaps were coded as missing. An estimate of net evolutionary divergence was calculated between the five Southeastern *Reticulitermes* using MEGA 5 by determining the number of base substitutions per site. The sequence data were used to infer optimal phylogenetic trees employing the following tree estimation methods as implemented by the listed software package: Maximum Likelihood (PHYML) and Maximum Parsimony (MEGA 5) [26,27,28,29,30,31,32]. ML (Maximum Likelihood) analysis for COI and COII was performed with PHYML 3.0 on the Phylogeny.fr: Robust Phylogenetic Analysis For The Non-Specialist [31]web server using the GTR+G+I model. The MP (Maximum Parsimony) analysis of COI and COII sequence used MEGA 5 with Close-Neighbor-Interchange (CNI) search and 1000 bootstrap replicates. Graphical representations of the resulting trees were improved using FigTree [32].

**Table 3:** Primer sequences used for amplification and gene sequencing.

Gene of interest	Primer pair (forward and reverse)	Sequences (5’→3’)
COII (685 bp)	TL2-J-3037	ATG GCA GAT TAG TGC AAT GG
	TK-N-3785	GTT TAA GAG ACC AGT ACT TG
COI (~800 bp) (partial sequence)	C1-J-2195	TTG ATT CTT TTG GTC ACT CCA TGA AGT
	TL2-N-3014	TCC TAA TTG CAC TTA ATC TGC CAT ATT

Phylogenetic relationships of *Reticulitermes* from the southeastern USA were compared to GenBank sequences from other regions of the world to support the hypothesis that the new species was not previously described. Primary molecular voucher specimens were deposited at the University of Georgia Collection of Arthropods (UGCA), Georgia Museum of Natural History, Athens, Georgia, USA. DNA extraction vouchers were deposited in HSERP Laboratory, University of Georgia, Athens, GA, USA. 

## 3. Results and Discussion

### 3.1. Morphological Characters

The new species had the smallest range of measurements for both alate and soldier samples for all morphological characters examined with the exception of soldier head capsule ratio (*sl*:*sw*), and soldier right mandible angles of curvature (*smac1* and *smac2*) (Table 4a). The range of *sl*:*sw* was similar to *R. virginicus*, and *smac1 *and *smac2* were similar to *R. flavipes *(Table 4a). 

The range of soldier head capsule measurements for the new species was 1.14–1.72 mm for *sl* and 0.70–0.99 mm for *sw* (Table 4a). The *sl*:*sw* for soldier head capsules ranged from 1.52–1.98 (Table 4a). The range for the soldier right mandible angle of curvature was 7.2–14.6° for *smac1* and 24.1–34.0° for *smac2* (Table 4a). Alate body length without wing (*abl*) ranged from 3.26–4.63 mm, and alate body length including wing (*ablw*) ranged from 6.53–7.88 mm (Table 4b). The length of average forewings (*afw*) was slightly longer than the average hind wings (*ahw*), ranging from 4.94–5.98 mm, and 4.81–6.21 mm, respectively (Table 4b).

### 3.2. Behavior

Alate samples were collected from Sapelo Island on 12 May 2005, 6 February 2007 and 6 March 2007. All samples of fully developed, sclerotized and winged, alates were collected directly from sampling devices prior to flight. We predict that flights of the new species would occur during the same time frame as the alates were fully sclerotized and winged.

Table 4aList of soldier characters measured by species showing mean, standard deviation, range of values and range of mean ± one std. dev.

**Table:** 

Soldier head capsule															
Species	Length (*sl*), mm					Width (*sw*), mm					Ratio of length: width (*sl:sw*)				
	Mean	Std. dev.	Min. ^a^	Max. ^b^	Range of mean ± 1	Mean	Std. dev.	Min. ^a^	Max. ^b^	Range of mean ± 1	Mean	Std. dev.	Min. ^a^	Max. ^b^	Range of mean ± 1
*R. flavipes*	1.693	0.119	1.21	1.91	1.57–1.81	1.044	0.074	0.73	1.17	0.97–1.12	1.625	0.084	1.43	1.83	1.54–1.71
*R. virginicus*	1.625	0.068	1.37	1.84	1.56–1.69	0.920	0.039	0.76	1.01	0.88–0.96	1.767	0.063	1.56	1.95	1.70–1.83
*R. hageni*	1.434	0.092	1.16	1.59	1.34–1.53	0.862	0.030	0.75	0.91	0.82–0.90	1.656	0.073	1.44	1.78	1.58–1.73
*R. malletei*	1.490	0.058	1.33	1.64	1.43–1.55	0.879	0.029	0.78	0.95	0.85–0.91	1.695	0.066	1.52	1.87	1.63–1.76
*R. nelsonae*	1.407	0.127	1.14	1.72	1.28–1.42	0.784	0.054	0.70	0.99	0.73–0.84	1.793	0.092	1.52	1.98	1.70–1.89

**Table:** 

Soldier mandible angle of curvature										
Species	*smac1*, °					*smac2*, °				
	Mean	Std. dev.	Min. ^a^	Max. ^b^	Range of Mean ± 1	Mean	Std. dev.	Min. ^a^	Max. ^b^	Range of Mean ± 1
*R. flavipes*	10.61	2.089	7.3	15.1	8.0–13.0	27.29	2.730	22.1	31.4	25.0–30.0
*R. virginicus*	13.62	1.719	11.7	18.1	12.0–15.0	32.60	2.003	29.4	36.7	31.0–35.0
*R. hageni*	8.39	1.199	6.9	11.2	7.0–10.0	23.39	1.307	21.5	27.3	22.0–25.0
*R. malletei*	10.51	1.366	8.1	13.2	9.0–12.0	25.85	2.282	22.4	32.9	24.0–28.0
*R. nelsonae*	10.66	2.206	7.2	14.6	8.0–13.0	27.27	2.654	24.1	34.0	25.0–30.0

Note: Shaded boxes represent values that were used in building the dichotomous key for soldiers and alates.

Table 4bList of alate characters measured by species showing mean, standard deviation, range of values and range of mean ± one std. dev.

**Table:** 

Species	Body (*abl*), mm					Body-wing (*ablw*), mm				
	Mean	Std. dev.	Min. ^a^	Max. ^b^	Range of mean ± 1	Mean	Std. dev.	Min. ^a^	Max. ^b^	Range of mean ± 1
*R. flavipes*	4.783	0.383	3.77	5.83	4.40–5.17	8.973	0.402	8.05	9.94	8.57–9.38
*R. virginicus*	4.021	0.214	3.56	4.44	3.81–4.24	7.414	0.213	6.89	7.90	7.20–7.63
*R. hageni*	4.083	0.323	3.41	5.35	3.76–4.41	7.810	0.318	7.25	8.64	7.49–8.13
*R. malletei*	4.023	0.302	3.53	4.99	3.72–4.33	8.238	0.394	6.91	9.28	7.84–8.63
*R. nelsonae*	3.928	0.236	3.26	4.63	3.69–4.16	7.080	0.291	6.53	7.88	6.79–7.37

**Table:** 

Species	Forewing (*afw*), mm					Hind wing (*ahw*), mm				
	Mean	Std. dev.	Min. ^a^	Max. ^b^	Range of mean ± 1	Mean	Std. dev.	Min. ^a^	Max. ^b^	Range of mean ± 1
*R. flavipes*	6.810	0.331	5.97	7.74	6.48–7.14	6.550	0.332	5.70	7.44	1.29–1.35
*R. virginicus*	5.532	0.193	5.15	6.05	5.34–5.73	5.418	0.193	4.78	5.78	1.32–1.37
*R. hageni*	5.965	0.263	5.47	6.52	5.70–6.23	5.739	0.257	5.24	6.19	1.28–1.34
*R. malletei*	6.375	0.328	5.13	7.31	6.04–6.70	6.100	0.339	4.92	6.95	1.27–1.32
*R. nelsonae*	5.430	0.212	4.94	5.98	5.22–5.64	5.315	0.297	4.81	6.21	1.27–1.31

Note: Shaded boxes represent values that were used in building the dichotomous key for soldiers and alates.

### 3.3. Dichotomous Key

Keys to the soldiers and alates of the *Reticulitermes* species of Georgia, USA, were constructed (Appendix 1, Appendix 2, respectively) based on values shown in Table 4a-Table 4b in shaded boxes representing the mean ± 1 std. dev. values for each character, with the exception of *sl:sw*. A minimum of 9 soldier head capsules are recommended to obtain a 95% confidence level for correct identification of *R. flavipes*, *R. virginicus*, and the new species, and 29 specimens for separating *R. malletei* from *R. hageni*. The minimum number of alate specimens recommended to obtain a 95% confidence level for discriminating all five species is 6. A more detailed discussion of the statistical analyses and generation of minimum sample size can be found in Lim (2011) [29].

### 3.4. Molecular Data

**Figure 4:**
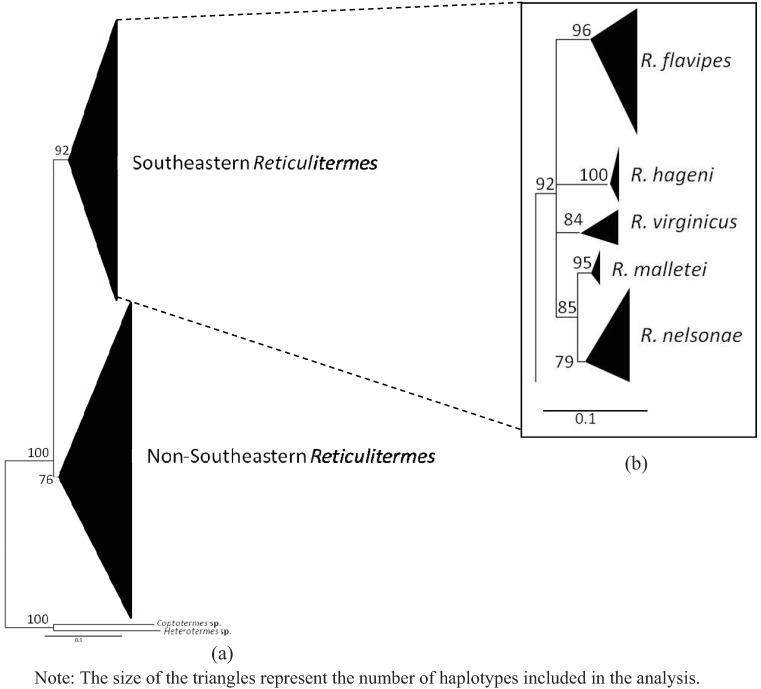
Maximum Likehood (ML) estimate of the *Reticulitermes* phylogeny based on mitochondrial cytochromeoxidase II (COII) gene data (665 bp). The scale bar represents 0.1 substitution/ site. (**a**) Collapsed tree topology illustrating the branch support and clades formed by *Reticulitermes* species from the southeastern USA and other regions of the world. (**b**) Collapsed tree topology illustrating the branch support and clades formed by *Reticulitermes* from southeastern USA.

**Figure 5:**
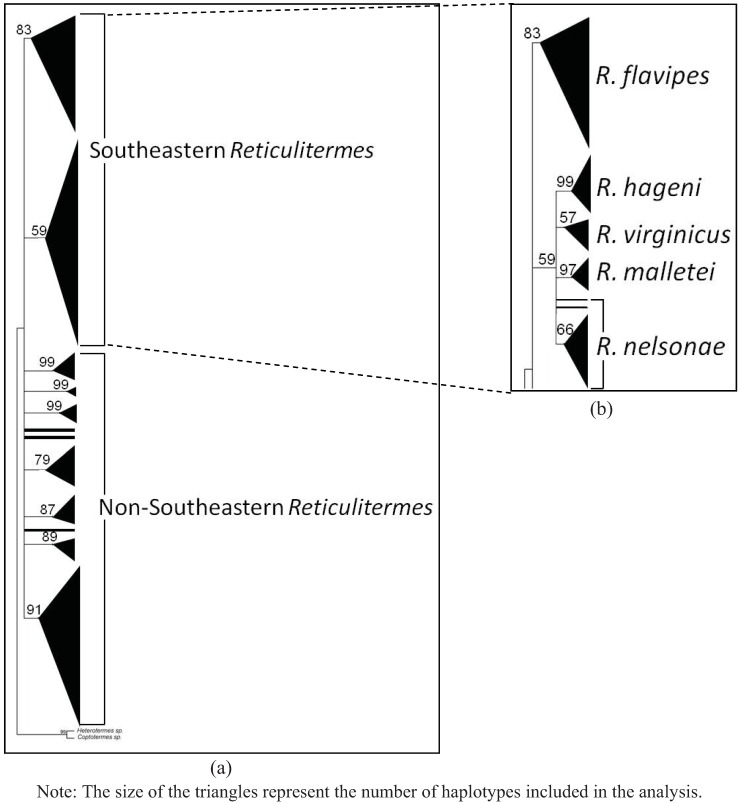
Maximum parsimony (MP) estimate of the *Reticulitermes* phylogeny based on mitochondrial ribosomal gene COII (665 bp) data. (**a**) Collapsed tree topology illustrating the branch support and clades formed by *Reticulitermes* species from the southeastern USA and other regions of the world. (**b**) Collapsed tree topology illustrating the branch support and clades formed by *Reticulitermes* from southeastern USA.

**Figure 6:**
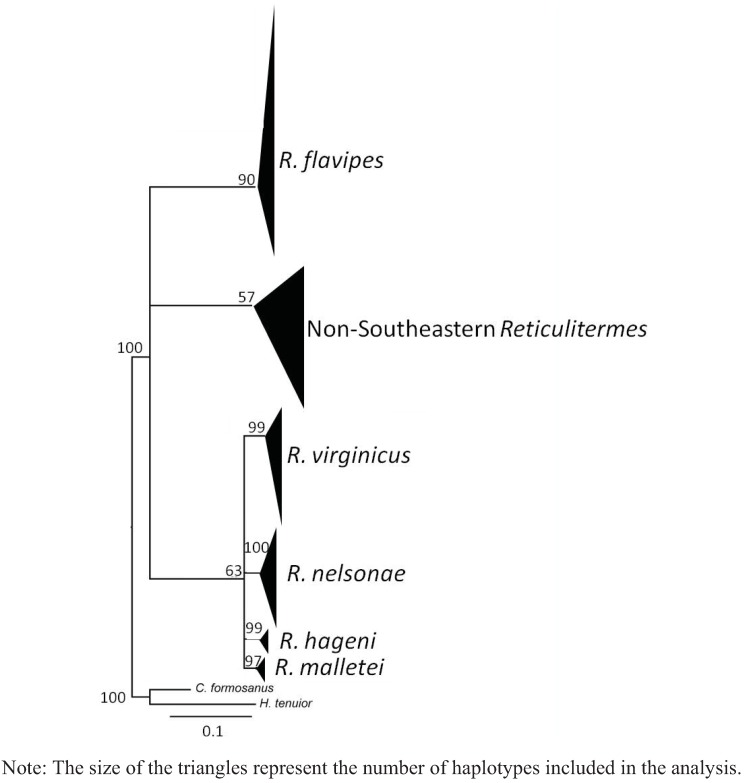
Maximum likelihood (ML) estimate of *Reticulitermes* phylogeny based on mitochondrial ribosomal gene COI (767 bp) data. Collapsed tree topology illustrating the branch support and clades formed by *Reticulitermes*.

**Figure 7:**
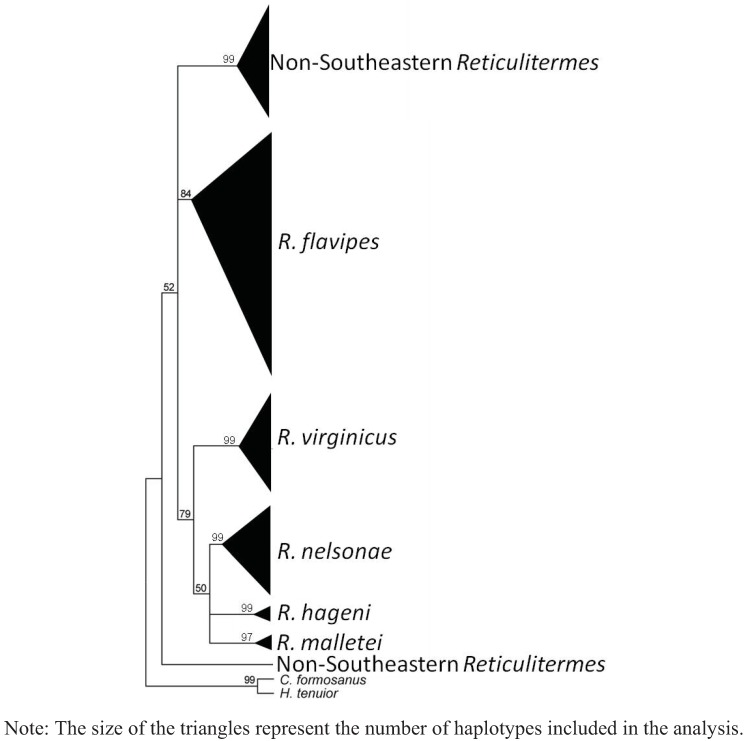
Maximum parsimony (MP) estimate of the *Reticulitermes* phylogeny based on mitochondrial ribosomal gene COI (767 bp) data. Collapsed tree topology illustrating the branch support and clades formed by *Reticulitermes*.

A total of 102 COII and 49 COI sequences were analyzed providing 20 new haplotypes for COII and 21 new haplotypes for COI. The estimated net evolutionary divergence among the five Southeastern *Reticulitermes* is shown in Table 5a and Table 5b for COII and COI respectively. The values indicate that *R. nelsonae* evolutionary divergence is comparable to the net evolutionary divergence observed between other described species from that region (Table 5a and Table 5b). Similar clades were recovered in all phylogenies from the COII and COI sequence data (Figure 4, Figure 5, Figure 6 and Figure 7). There are five reference sequences (labeled as α in Figure S1, Figure S2, Figure S3 and Figure S4) obtained from specimens where both alates and soldiers from that same collection point and date matched the described morphological criteria for the respective species (Figure 4, Figure 5, Figure 6 and Figure 7). Two sequences in the *R. malletei* clade for COII and COI were labeled β (Figure S1, Figure S2, Figure S3 and Figure S4) to signify that these sequences were corroborated with the original 16SrRNA haplotype sequences in Austin *et al.* (2007). 

**Table 5a:** Estimate of net evolutionary divergence between species for COII.

Species	*R. flavipes*	*R. virginicus*	*R. hageni*	*R. malletei*	*R. nelsonae*
*R. flavipes*					
*R. virginicus*	0.052				
*R. hageni*	0.047	0.033			
*R. malletei*	0.046	0.036	0.031		
*R. nelsonae*	0.059	0.049	0.044	0.039	

**Table 5b:** Estimate of net evolutionary divergence between species for COI.

Species	*R. flavipes*	*R. virginicus*	*R. hageni*	*R. malletei*	*R. nelsonae*
*R. flavipes*					
*R. virginicus*	0.051				
*R. hageni*	0.048	0.040			
*R. malletei*	0.042	0.037	0.027		
*R. nelsonae*	0.050	0.044	0.036	0.029	

The COII alignment included 665 bp of the 685 bp full length to size-match the length of some sequences retrieved from GenBank. The comparisons yielded 216 variable sites, of which 178 were parsimony-informative. Maximum likelihood (ML) and maximum parsimony (MP) were employed to construct phylogenetic trees that included selected GenBank sequences (as of 8 March 2011) for *Reticulitermes*. All inferred phylogenetic trees were well resolved for the Southeastern *Reticulitermes* showing consistent and strongly corroborated topologies with the exception of two haplotypes in the *R. nelsonae* clade (JF796236 and AF525328) from the MP phylogeny that are recovered as polytomies containing *R. nelsonae*, *R. malletei*, *R. virginicus* and *R. hageni* (Figure 4, Figure 5, Figure S1 and Figure S2). The inferred ML tree shows high branch support (>84) for all species clades within the Southeastern *Reticulitermes* group with Ln likelihood = −5075.935 (Figure 4). The MP analysis resulted in 42 most parsimonious trees (length = 854, CI = 0.33, RI = 0.82) (Figure 5). The composite index for all sites = 0.300, and a composite index for parsimony-informative sites = 0.270 that used 1000 bootstraps to generate the consensus tree (Figure 5). COII sequences were deposited in GenBank (EU689013, JF796229-JF796233, and JF796235-JF796236) and are listed in bold in the supplemental phylogenies (Figure S1 and Figure S2). The ML and MP phylogeny for COII showed separation between *Reticulitermes* from the southeastern USA and *Reticulitermes* from other regions (Figure 4 and Figure 5). 

The alignment of COI included 767 bp of the 801 bp partial length to size-match with some of the GenBank sequences retrieved for this analysis, Results showed 146 variable sites of which 113 were parsimony-informative. Inferred ML tree shows high branch support for most clades with Ln likelihood = −2905.620 (Figure 6). MP analysis resulted in 33 most parsimonious trees of 366 steps with a consistency index = 0.556, a retention index = 0.892, a composite index for all = 0.565, and a composite index for parsimony-informative sites = 0.496 that used 1000 bootstraps to generate the consensus tree that was similar in tree topology to the ML analysis (Figure 6 and Figure 7). COI sequences also were deposited in GenBank (JN207486-JN207491) and are listed in bold in the supplemental phylogenies (Figure S3 and Figure S4).

The phylogenetic trees from ML and MP analyses using the COI data also recovered genetic separation between *Reticulitermes* from the southeastern region of the USA and *Reticulitermes* from other parts of the world (Figure 6 and Figure 7). No COI sequences were found in GenBank for *Reticulitermes* from the western USA.

## 4. Systematics

**Figure 8:**
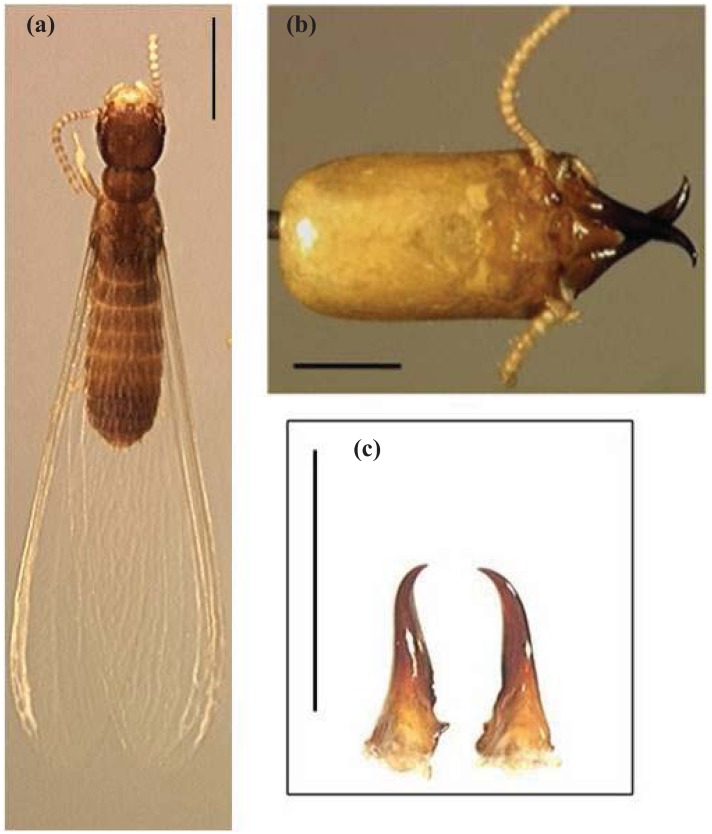
*Reticulitermes nelsonae, *diagnostic morphological characters for species identification: (**a**) Alate, dorsal, scale bar = 1.0 mm, (**b**) Soldier head capsule, dorsal, scale bar = 0.5 mm, and (**c**) Soldier mandible pair, dorsal, scale bar = 1.0 mm.

**Figure 9:**
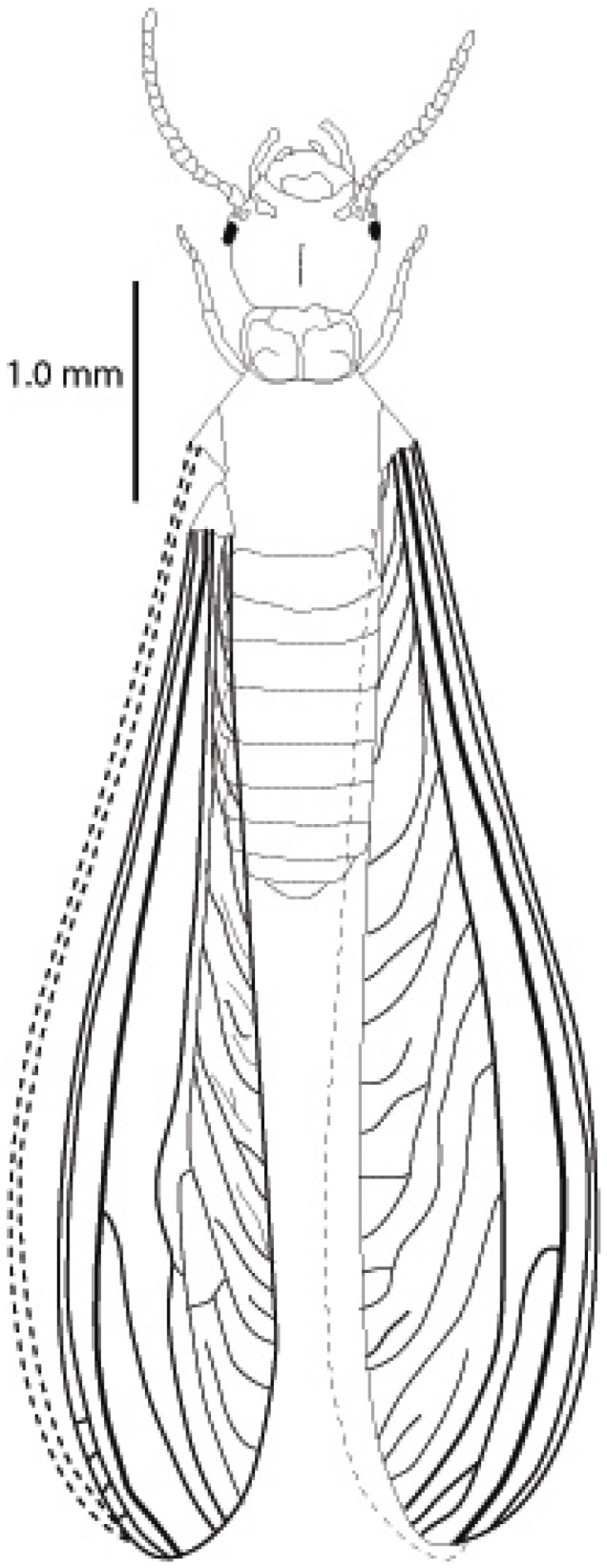
Habitus drawing of *R. nelsonae* alate, showing fore- and hind wing along with wing venation. Scale bar is 1.0 mm.

**Figure 10:**
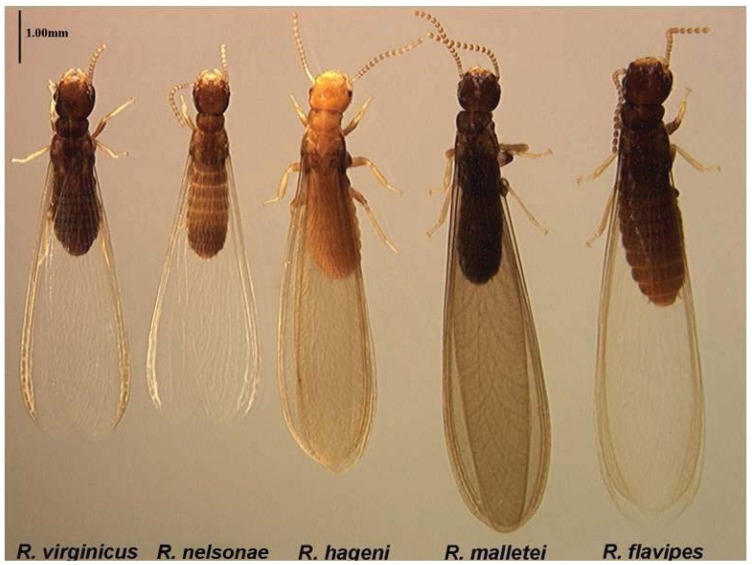
*Reticulitermes* species alates from the southeastern USA. From left to right: *R. virginicus*, *R. nelsonae*, *R. hageni*, *R. malletei*, *R . flavipes*. Scale bar = 1.00 mm.

*Reticulitermes nelsonae* Lim and Forschler, new species

(Figure 1, Figure 3, Figure 8, Figure 9 and Figure 10)

HOLOTYPE (alate, ♀): “USA: Georgia, McIntosh Co., Sapelo Island, 31°23'43.32"N 

81°16'38.23"W, 6.II .2007, D. Sillam-Dussès” (AMNH).

ALLOTYPE (alate, ♂): “USA: Georgia, McIntosh Co., Sapelo Island, 31°23'43.32"N 

81°16'38.23"W, 6. II. 2007, D. Sillam-Dussès” (AMNH).

PARATYPES (alate): USA: Georgia: McIntosh Co., Sapelo Island, 31°23'43.32"N

81°16'38.23"W, 6.II.2007, D. Sillam-Dussès (1 ♂, 1♀, UGCA; 1 ♂, 1 ♀, NMNH).

PARATYPES (soldier): USA: Georgia: McIntosh Co., Sapelo Island, 31°23'43.32"N

81°16'38.23"W, 6.II.2007, D. Sillam-Dussès (1 ♂, 1♀, UGCA; 1 ♂, 1 ♀, NMNH).

PARATYPES (worker): USA: Georgia: McIntosh Co., Sapelo Island, 31°23'43.32"N

81°16'38.23"W, 6.II.2007, D. Sillam-Dussès (1 ♂, 1♀, UGCA; 1 ♂, 1 ♀, NMNH).

### 4.1. Diagnosis

*Soldier* (Table 4a, Figure 8b and 8c): Head capsule small (mean *sl* 1.407 mm, mean *sw* 0.054 mm, mean *sl:sw* 1.793 mm) (Table 4a). 

*Alate* (Table 4b, Figure 8a, Figure 9 and Figure 10): Body length, with (*ablw*) and without (*abl*) wing, small (mean *abl* 3.93 mm, mean *ablw* 7.080 mm), body color pale brown and wings non-pigmented (Figure 8a, Figure 9 and Figure 10). 

### 4.2. Description

There is little sexual dimorphism for soldier or alates, males and females can however, be differentiated by the form of the 8th sternal plate (Zimet & Stuart, 1982).

*Soldier* Head capsule rectangular in shape, typically longer than wide (Figure 8b). Head capsule color yellowish with dark brown to black mandibles (Figure 8b). Body color light yellowish to white. Mean head capsule length (*sl*) 1.41 mm ± 0.13 (Table 4a), mean width (*sw*) 0.78 mm ± 0.05 (Table 4a), mean ratio length-to-width (*sl : sw*) 1.793 ± 0.09 (Table 4a). Mean mandible curvature angles: *smac1 *= 10.7° ± 2.21, *smac2 *= 27.27° ± 2.65 (Table 4a). 

*Alate* Body color pale brown with 14 antennal segments (Figure 8a and Figure 10). Wings non-pigmented (Figure 8a and Figure 10). Legs light to dark brownish with 14 antennal segments (Figure 8a and Figure 10). Mean body length without wing (*abl*) 3.93 mm ± 0.24 (Table 4b). Mean body length with wings (*ablw*) 7.08 mm ± 0.29 (Table 4b). Mean forewing length (*afw*) 5.43 mm ± 0.21. Mean hind wing length (*ahw*) 5.32 mm ± 0.30 (Table 4b).

### 4.3. Etymology

This patronym was established to honor Lori J. Nelson (USDA Forest Service, Buchanan, CA, USA) who realized in 1996 that specimens collected on Sapelo Island, a barrier island off the Atlantic coast of Georgia, were notably different from all previously described *Reticulitermes* species based on analysis of cuticular hydrocarbons [17,18,19].

*Behavior*

*Reticulitermes nelsonae* is expected to swarm from February to May. *Reticulitermes hageni* swarms from August to October. *Reticulitermes flavipes* flights have been recorded from November through April thus overlapping with *R. nelsonae *[29]. *Reticulitermes nelsonae *flight times also overlap with *R. virginicus* and *R. malletei*, both of which have been recorded in May [10,13,33].

### 4.4. Distribution

**Figure 11:**
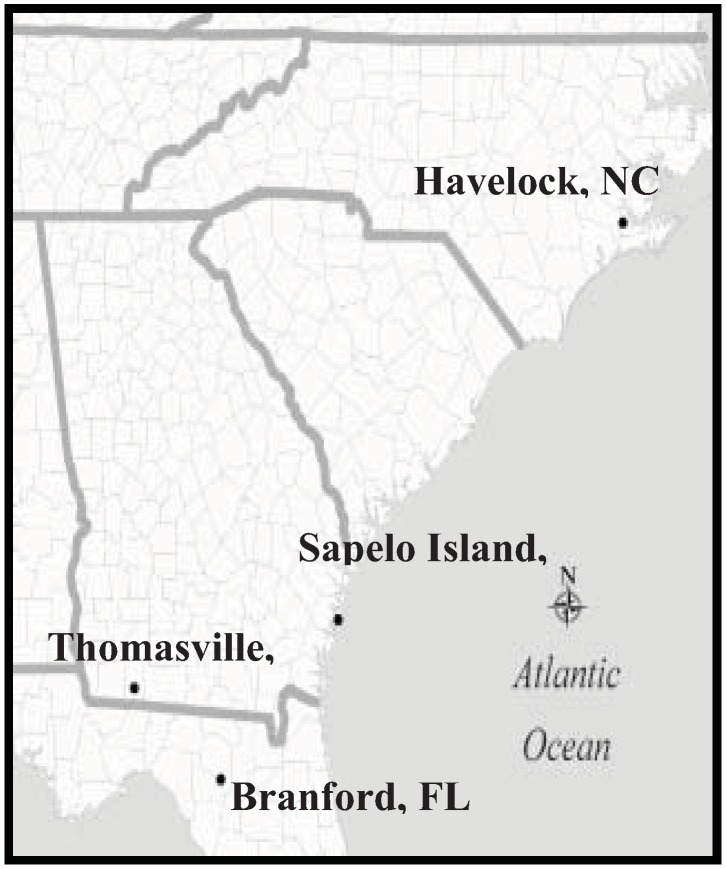
Distribution map of locales where the new species, *Reticulitermes nelsonae* have been collected*.*

*Reticulitermes nelsonae* is found in the southeastern region of the United States, in the Atlantic Coastal Flatwoods and South Coastal Plain soil provinces (Table 2, Figure 11). The species has not been detected in the Piedmont soil province despite sampling in that region. In addition to the type locality on Sapelo Island, GA, this species has been collected in Croatan National Forest in Havelock, NC, Greenwood Plantation in Thomasville, GA, and Branford, FL (Table 2, Figure 11).

### 4.5. Comments/Remarks

*Soldier Reticulitermes nelsonae* head capsule length (*sl*) is at least 0.2 mm shorter than *R. flavipes* and *R. virginicus *(Table 4a). *Reticulitermes nelsonae* head capsule width (*sw )* at least 0.1 mm smaller than *R. flavipes* and *R. virginicus *(Table 4a). The *sl* and *sw* are also more than 0.1 mm smaller than *R. malletei* (Table 4a). The *R. nelsonae smac2* is more than 24° (typically 24°–30°), while *R. hageni smac2* is less than 25° (typically 22°–25°) (Table 4a). 

*Alate Reticulitermes nelsonae abl *and *ablw* are typically 3.7 mm–4.2 mm and 6.8 mm–7.4 mm, whereas in *R. flavipes *those same characters are more than 4.4 mm and 8.6 mm, respectively (Table 4b). *Reticulitermes nelsonae afw* and *ahw* are 1.0 mm shorter than *R. flavipes *(Table 4b). Body color differs from that of *R. hageni’s *yellowish to yellowish-brown body color (Figure 10). *Reticulitermes nelsonae *wings are non-pigmented, while *R. malletei* has pigmented wings (Austin *et al.*, 2007) and forewing (*afw*) measurements are 0.4 mm shorter than *R. malletei*. *Reticulitermes nelsonae* and *R. virginicus* share similar morphometric ranges, but differ in body color, with *R. nelsonae *having light brown color and *R. virginicus* dark brown. The ratio of mean body length including wings to mean forewing length (*ablw*: *afw*) is typically 1.27–1.31 for *R. nelsonae*, while for *R. virginicus* it is 1.32–1.37 (Table 4b).

### 4.6. Genetics

Sequences from cytochrome oxidase I (COI) and cytochrome oxidase II (COII) genes generated data that showed *R. nelsonae* was genetically unique as its haplotypes consistently formed a separate clade from the other described species (Figure 4, Figure 5, Figure 6 and Figure 7).

## 5. Discussion

The genus *Reticulitermes* is in need of a thorough taxonomic revision, so describing new species is a task beset with numerous difficulties. Broad intraspecific morphological variation exacerbates the issue of species discrimination, yet multiple independent lines of evidence (listed below) support recognition of *R. nelsonae *as a new species. The description of a single new species within taxa of economic and ecological significance, like *Reticulitermes*, is justifiable outside of the context of an exhaustive revision. It is our hope that this work will serve as a foundation toward a revision of *Reticulitermes*.

### 5.1. Cuticular Hydrocarbon

An examination of Haverty *et al.* (1999) [18] and Jenkins *et al.* (2000) [19] indicates that the hydrocarbon phenotypes GA-L and GA-I most likely correspond to *R. nelsonae*. Jenkins *et al.* (2000) [19] observed that two of their collections were “different morphologically, chemically and genetically”. We now believe these samples, identified as haplotypes BH25 (JF796235) and HH11 (JF796236), are *R. nelsonae* because we recovered those haplotypes in the *R. nelsonae* clade for the COII gene (haplotypes are underlined in Figure S1 and Figure S2). We, therefore, reason that the cuticular hydrocarbon phenotypes GA-L and GA-I reported by [17,18] belong to *R. nelsonae*. 

### 5.2. Morphology

Morphological separation of *Reticulitermes* species is notoriously difficult. Our morphometric measurements provided a range of overlap consistent with past reports for the genus [13,14,16,17,21,34]. Dichotomous keys for soldiers and alates of *Reticulitermes* species collected in Georgia (Appendix 1 and Appendix 2) were prepared to distinguish the five species endemic to the southeastern USA. The measurements used to build the soldier key can separate *R. nelsonae* soldiers from all previously described species with the exception of *R. hageni*, which overlap on all measures at the upper range for *R. nelsonae* (Table 4a and Appendix 1). Alates of all species can be separated based on the combination of body color, morphometric measurements, and flight times (Table 4b and Appendix 2). 

The first author has prepared a more extensive study of the literature on morphological variation in *Reticulitermes* species from the southeastern USA [29]. We recommend that 6 alates and/or 29 soldiers specimens be used to achieve a 95% confidence in morphometric-based species diagnosis [29]. 

### 5.3. Genetics

Congruent and similar phylogenies were obtained from ML and MP analyses for both COII and COI sequences (Figure 4, Figure 5, Figure 6 and Figure 7). Genetics of *Reticulitermes* from the southeastern USA were consistently differentiated from that of *Reticulitermes* from other regions of the world and haplotypes of *Reticulitermes nelsonae *were further differentiated within the ‘Southeastern *Reticulitermes*’ grouping and thus are genetically unique from previously described *Reticulitermes* (Figure 4, Figure 5, Figure 6 and Figure 7). The net evolutionary distance dataset demonstrates that the ‘genetic uniqueness’ of *R. nelsonae* is within the range of the already described species (Table 5a and Table 5b).

Three *Reticulitermes* species (*R. virginicus*, *R. hageni* and *R. malletei*) from the southeastern USA have clear separation between COII haplotype designations within their respective species clades, while mixed haplotype designations were observed in the *R. flavipes* and *R. nelsonae* clade (see supplementary Figure S1 and Figure S2). The *R. flavipes* clade includes *R. santonensis* (which has been synonymized as *R. flavipes*) and *R. arenincola *haplotypes (Figure S1 and Figure S2). Previous reports on *R. arenincola* support our findings showing *R. arenincola* is genetically similar to *R. flavipes* [35,36,37].The *R. nelsonae* clade includes four GenBank sequences that were designated as *R. hageni* (NC009501, AY808088, AY808089, and AF525328) (Figure S1 and Figure S2). Voucher specimens for these GenBank accessions have been requested and to date two examined (AY808088, AY808089). The mistaken GenBank accessions are most likely the result of the fact that only 3 of the 5 species endemic to the southeastern USA are listed in published keys and the available taxonomic keys would have identified *R. nelsonae* specimens as *R. hageni* [13,14,15].

The 767 bp COI gene phylogenies also indicate that haplotypes of *Reticulitermes* species from the southeastern USA are different from *Reticulitermes* found in other areas of the USA (Figure 4, Figure 5, Figure 6 and Figure 7). *Reticulitermes santonensis* was again recovered within the *R. flavipes* clade further supporting junior synonym status (Figure S3 and Figure S4). One GenBank *R. hageni* sequence (EF 206320) was recovered within the *R. nelsonae* clade (Figure S3 and Figure S4) which is not surprising, and as mentioned, the specimen would have been identified as *R. hageni* based on published keys [13,14,15]. 

Molecular phylogenies are an estimation of plausible species relationships and therefore detailed research and comparison is warranted to accurately identify species designations for gene sequence data. Cytochrome oxidase II (COII), with a length of 685 bp, has been a valuable marker for identification of *Reticulitermes* species [19,22,38,39] as evidenced by Sillam-Dusses and Forschler (2010) who reported an undescribed species based on genetics alone [22]. We recovered *R. arenincola* Goellner 1931 in the *R. flavipes* clade lending support for synonymy with *R. flavipes* (Figure 4 and Figure 5) [12,35,36,37,40]. 

We suggest that future genetic analysis provide reference sequences for COII and COI genes that are from specimens corroborated with morphological descriptions for *Reticulitermes* species (as denoted by α in Figure S1, Figure S2, Figure S3 and Figure S4).

## 6. Conclusions

We echo past recommendations that species discrimination based on morphology should include data from both alates and soldiers, from the same collection, for accurate identification [13,14]. A prominent conclusion from this study is that *Reticulitermes* species discrimination should be attempted using morphometric characters from both castes accompanied by genetic and or other chemical evidence [21,41,42,43]. Based on the data obtained in this study, *R. nelsonae* is a true entity that satisfies the following species concepts: the morphological [44], phylogenetic [44,45], genetic [46], ecological and reproductive isolation species concepts [44,47]. Further examination will determine if the distribution of *R. nelsonae* is restricted, as currently described to the Atlantic Coastal Flatwoods and South Coastal Plain soil provinces across the southeastern United States.

Additional studies of the morphological and haplotype diversity of *Reticulitermes* are needed throughout the distributional range. Data sets combining cuticular hydrocarbon phenotypes, genetics, and morphology, should be explored further to facilitate identification of species within this taxonomically challenging genus.

## Figures and Tables

**Supplementary 1:**
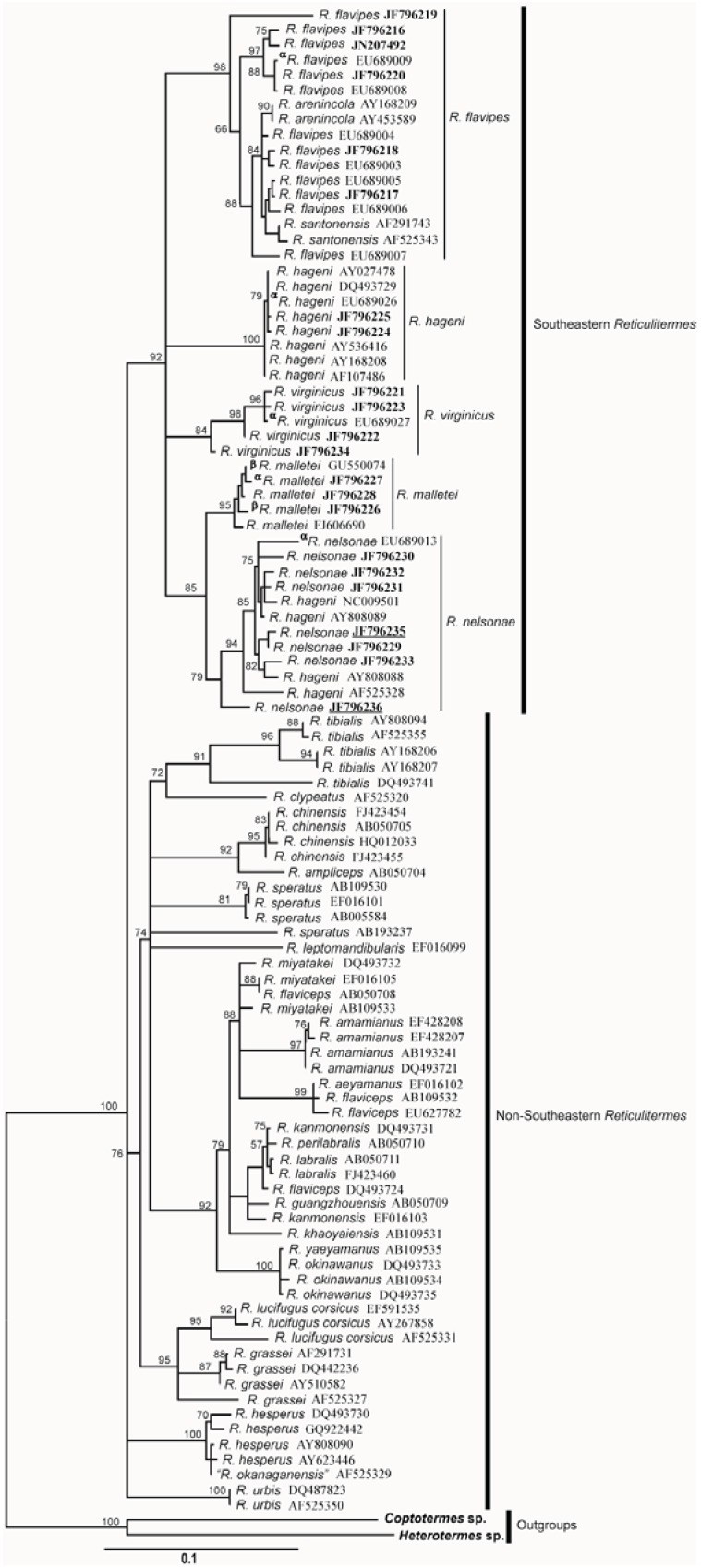
Maximum likelihood (ML) estimate of the *Reticulitermes* phylogeny based on mitochondrial ribosomal gene COII (665 bp) data. Bold Accession numbers indicate sequence from specimens matched with collections used in the morphometric portion of this study. Underlined Accession numbers are BH25 = JF796235, HH11 = JF796236.

**Supplementary 2:**
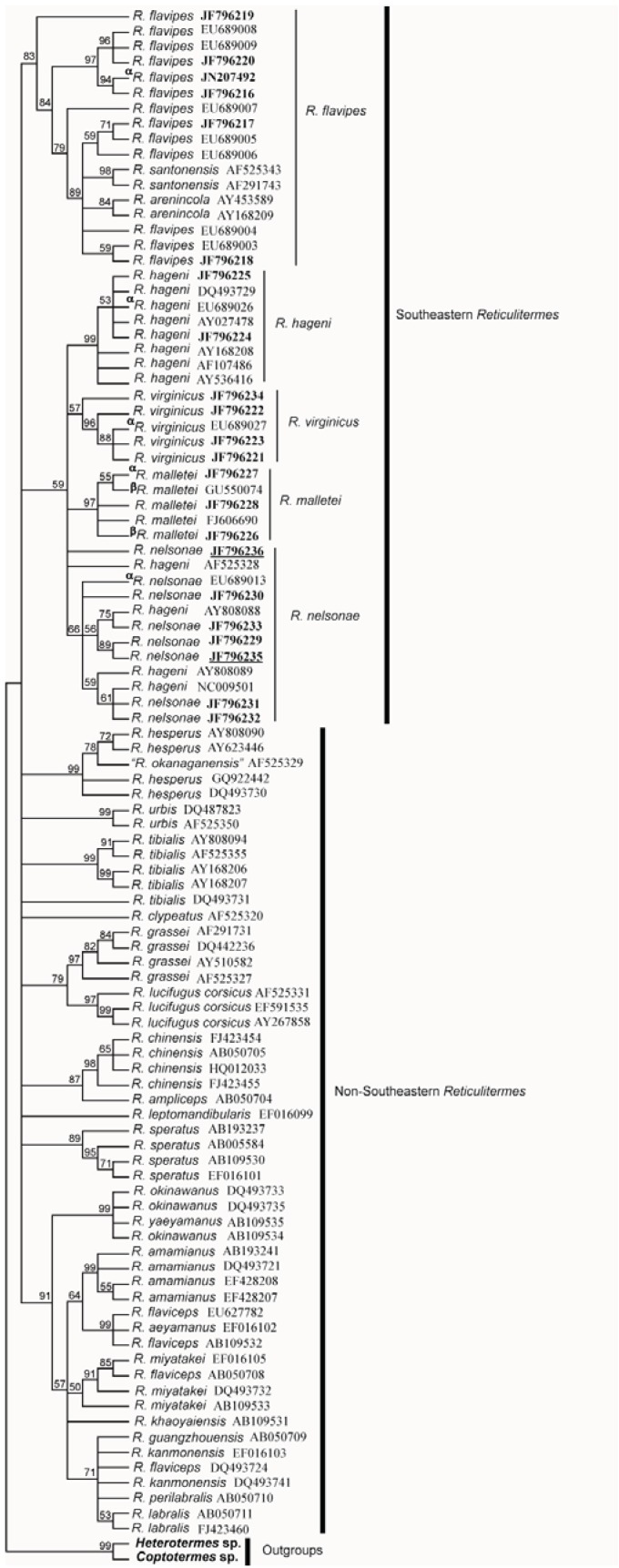
Maximum parsimony (MP) estimate of the *Reticulitermes* phylogeny based on mitochondrial ribosomal gene COII (665 bp) data. Bold Accession numbers indicate sequence from specimens matched with collections used in the morphometric portion of this study. Underlined Accession numbers are BH25 = JF796235, HH11 = JF796236.

**Supplementary 3:**
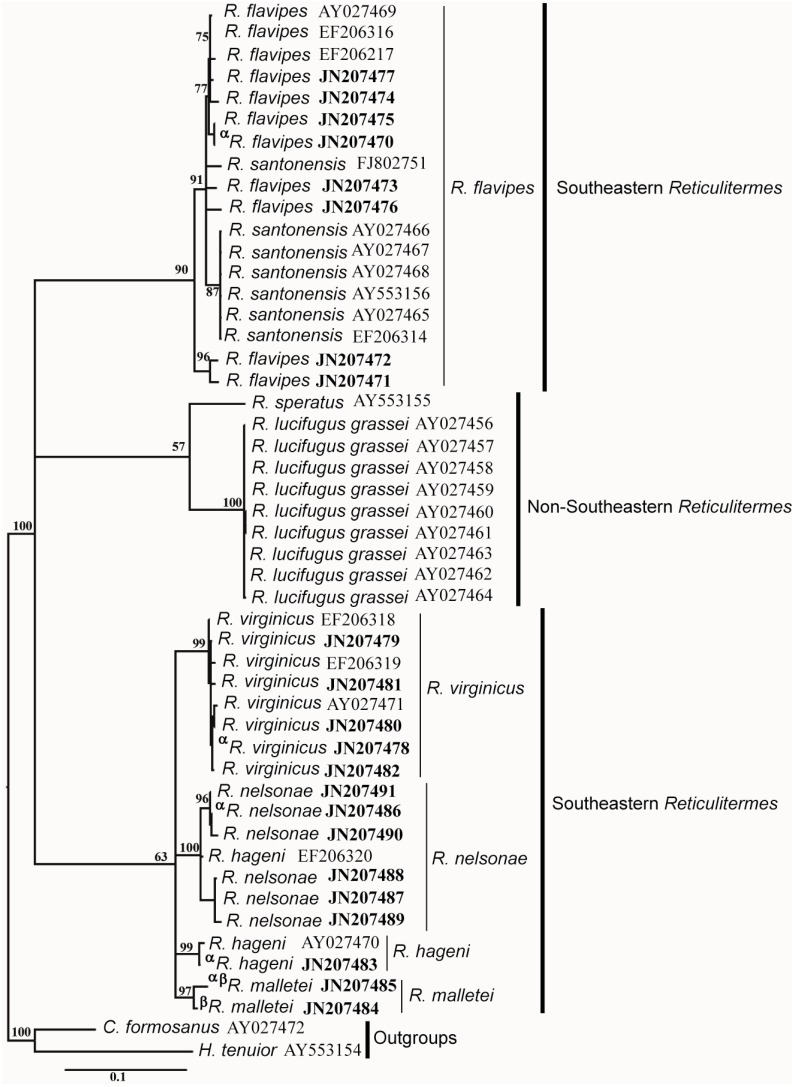
Maximum likelihood (ML) estimate of the *Reticulitermes* phylogeny based on mitochondrial ribosomal gene COI (767 bp) data.Bold Accession numbers indicate sequence from specimens matched with collections used in the morphometric portion of this study.

**Supplementary 4:**
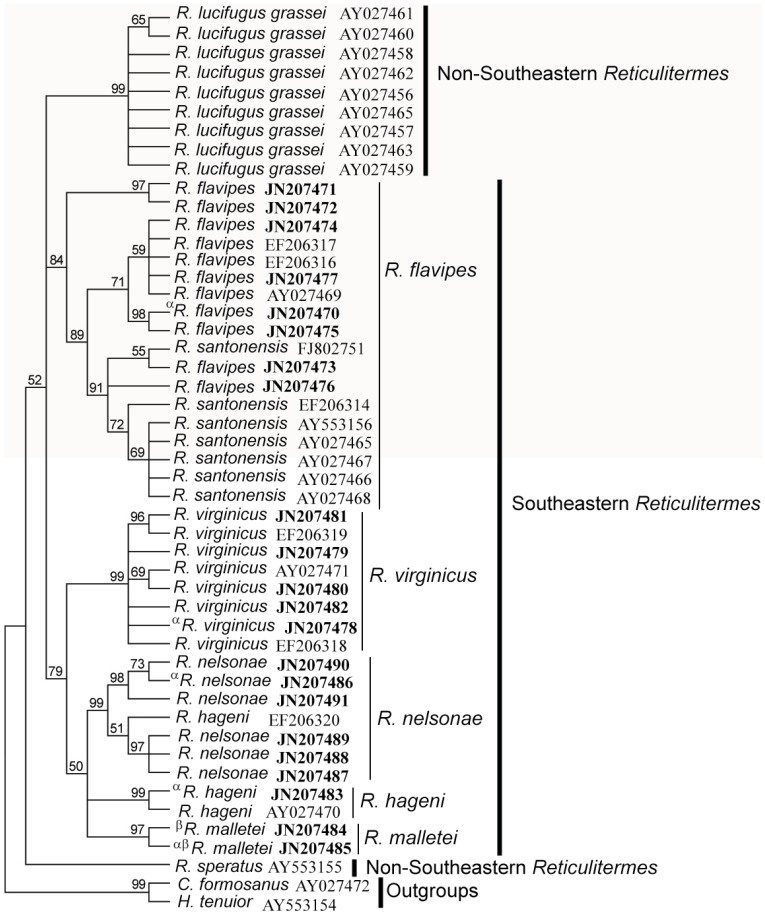
Maximum parsimony (MP) estimate of the *Reticulitermes* phylogeny based on mitochondrial ribosomal gene COI (767 bp) data. Bold Accession numbers indicate sequence from specimens matched with collections used in the morphometric portion of this study.

## Appendix 1: Key 1

1 Head capsule length without mandible (*sl*) typically ≥ 1.56 mm (Figure 1) ………… 2

- Head capsule length without mandible (*sl*) typically ≤ 1.55 mm (Figure 1) ………… 3

2 Head capsule width (*sw*) mean 0.92 mm typically 0.88–0.96 mm (Figure 1). Right mandible curvature angle (*smac2*) mean 32.6° typically ≥ 31° (31°–35°) (Figure 2) ……………….***R. virginicus***

- Head capsule width (*sw*) mean 1.0 mm typically 0.97–1.12mm (Figure 1). Right mandible curvature angle (*smac2*) mean 27.3° typically ≤ 25° (25°–30°) (Figure 2)..………………….………***R. flavipes***

3 Head capsule width (*sw*) mean 0.86 mm typically 0.82–0.90 mm (Figure 1).

Right mandible angle of curvature (*smac2*) mean 23.39° typically ≤ 25° (22°–25°) (Figure 2).

Head capsule length without mandible (*sl*) mean 1.43 mm typically 1.34–1.53 mm (Figure 1).

………………………………………………………………………………... ***R. hageni***

- Head capsule width (*sw*) mean 0.88 mm typically 0.85–0.91 mm (Figure 1).

Right mandible angle of curvature (*smac2*) mean 25.85° typically ≥ 25° (25°–30°) (Figure 2).

Head capsule length without mandible (*sl*) mean 1.49 mm typically ≥1.43 mm (1.43–1.55 mm) (Figure 1).……………………………………………………………………….. ***R. malletei***

- Head capsule width (*sw*) mean 0.78 mm typically 0.73 mm–0.84 mm (Figure 1).

Right mandible angle of curvature (*smac2*) mean 27.27° typically 24–28° (Figure 2). Head capsule length without mandible (*sl*) mean 1.41mm typically ≤1.42 mm (1.28–1.42 mm) (Figure 1).………………………………………………………………………………..***R. nelsonae***

## Appendix 2: Key 2

1- Body color light yellowish brown, pigmented wings (Figure 11). Swarm dates August–October

…………………………………………………………………………………………... ***R. hageni***

- Body color pale brown to dark brown, blackish, pigmented or non-pigmented wings (Figure 11).

Swarm dates November - May ….……………………………………………………. 2

2- Body length including wings (*ablw*) mean 8.97 mm typically ≥ 8.57 mm (8.57–9.38 mm) (Figure 6 and Figure 7). Body length without wings (*abl*) mean 4.78 mm typically ≥ 4.40 mm (4.40–5.17 mm) (Figure 6 and Figure 7)……………………………………………… *****R. flavipes*****

- Body length including wings (*ablw*) typically ≤ 8.57 mm (6.79–8.57 mm) (Figure 6 and Figure 7).

Body length without wings (*abl*) typically ≤ 4.41mm (3.69–4.41 mm) (Figure 6 and Figure 7)….3

3- Average forewing length (*afw*) mean 6.37 mm ≥ 6.04 mm (6.04–6.70 mm) (Figure 6 and Figure 7).

Average hind wing length (*ahw*) mean 6.10 mm typically ≥ 5.76 mm (5.76–6.44 mm) (Figure 6 and Figure 7)……………………………………………………………………………………***R. malletei***

- Average forewing length (*afw*) ≤ 5.70 mm (5.22–5.73 mm) (Figure 6 and Figure 7).

Average hind wing length (*ahw*) typically ≤ 5.61 mm (5.02–5.61 mm) (Figure 6 and Figure 7)…… 4

4-Body color dark brown to black, non-pigmented wings (Figure 10). Ratio of body length with wing (*ablw*) to average forewing (*afw*) typically 1.32–1.37. Swarm dates April–May ……. ***R. virginicus***

- Body color pale brown, non-pigmented wings (Figure 10). Ratio of body length with wing (*ablw*) to average forewing (*afw*) typically 1.27–1.31. Swarm season February–May….……… ***R. nelsonae***
