# Correction: Lim and Forschler, *Reticulitermes nelsonae*, a New Species of Subterranean Termite (Rhinotermitidae) from the Southeastern United States. Insects 2012, 3, 62-90.

**DOI:** 10.3390/insects3010131

**Published:** 2012-02-03

**Authors:** Su Yee Lim, Brian T. Forschler

**Affiliations:** Household and Structural Entomology Laboratory, Department of Entomology, 413 Biosciences Building, 120 Cedar St., University of Georgia, Athens, GA 30602, USA; E-Mail: bfor@uga.edu

Following publication of our article [1], we found errors in Table 4b. These errors do not make any difference to the main findings and conclusions reported in our paper. The numbers in the column for the range of mean ± 1 standard deviation for hind wings were incorrect. We wish to apologize for the mistake made and inconveniences caused. 

The corrected Table 4b is listed below:

**Table 4:** List of alate characters measured by species showing mean, standard deviation, range of values and range of mean ± one std. dev.

Species	Body (*abl*), mm					Body-wing (*ablw*), mm				
	Mean	Std. dev.	Min. ^a^	Max. ^b^	Range of mean ± 1	Mean	Std. dev.	Min. ^a^	Max. ^b^	Range of mean ± 1
*R. flavipes*	4.783	0.383	3.77	5.83	4.40–5.17	8.973	0.402	8.05	9.94	8.57–9.38
*R. virginicus*	4.021	0.214	3.56	4.44	3.81–4.24	7.414	0.213	6.89	7.90	7.20–7.63
*R. hageni*	4.083	0.323	3.41	5.35	3.76–4.41	7.810	0.318	7.25	8.64	7.49–8.13
*R. malletei*	4.023	0.302	3.53	4.99	3.72–4.33	8.238	0.394	6.91	9.28	7.84–8.63
*R.* *nelsonae*	3.928	0.236	3.26	4.63	3.69–4.16	7.080	0.291	6.53	7.88	6.79–7.37
**										
**Species**	**Forewing (*afw*), mm**					**Hind wing (*ahw*), mm**				
	**Mean**	**Std. dev.**	**Min. ^a^**	**Max. ^b^**	**Range of mean ± 1**	**Mean**	**Std. dev.**	**Min. ^a^**	**Max. ^b^**	**Range of mean ± 1**
*R. flavipes*	6.810	0.331	5.97	7.74	6.48–7.14	6.550	0.332	5.70	7.44	6.22–6.88
*R. virginicus*	5.532	0.193	5.15	6.05	5.34–5.73	5.418	0.193	4.78	5.78	5.23–5.61
*R. hageni*	5.965	0.263	5.47	6.52	5.70–6.23	5.739	0.257	5.24	6.19	5.48–6.00
*R. malletei*	6.375	0.328	5.13	7.31	6.04–6.70	6.100	0.339	4.92	6.95	5.76–6.44
*R.* *nelsonae*	5.430	0.212	4.94	5.98	5.22–5.64	5.315	0.297	4.81	6.21	5.02–5.61

Note: Shaded boxes represent values that were used in building the dichotomous key for soldiers and alates.
